# Epigenetic Regulation of Inflammatory Cytokine-Induced Epithelial-To-Mesenchymal Cell Transition and Cancer Stem Cell Generation

**DOI:** 10.3390/cells8101143

**Published:** 2019-09-25

**Authors:** Georgios S. Markopoulos, Eugenia Roupakia, Kenneth B. Marcu, Evangelos Kolettas

**Affiliations:** 1 Laboratory of Biology, School of Medicine, Faculty of Health Sciences, University of Ioannina, University Campus, 45110 Ioannina, Greece; gmarkop@cc.uoi.gr (G.S.M.); ev.roupakia@gmail.com (E.R.); 2 Biomedical Research Division, Institute of Molecular Biology and Biotechnology, Foundation for Research and Technology, 45115 Ioannina, Greece; kenneth.marcu@stonybrook.edu; 3 Biomedical Research Foundation of the Academy of Athens (BRFAA), 4 Soranou Ephessiou Street, 115-27 Athens, Greece; 4 Departments of Biochemistry and Cell Biology and Pathology, Stony Brook University, Stony Brook, NY 11794-5215, USA

**Keywords:** inflammatory cytokines, epithelial-to-mesenchymal cell transition (EMT), inflammatory cytokine-induced EMT (ICI-EMT), cancer stem cells (CSCs), tumor microenvironment (TME), transcription factors (TFs), epigenetic regulators, feedback regulatory loops

## Abstract

The neoplastic transformation of normal to metastatic cancer cells is a complex multistep process involving the progressive accumulation of interacting genetic and epigenetic changes that alter gene function and affect cell physiology and homeostasis. Epigenetic changes including DNA methylation, histone modifications and changes in noncoding RNA expression, and deregulation of epigenetic processes can alter gene expression during the multistep process of carcinogenesis. Cancer progression and metastasis through an ‘invasion–metastasis cascade’ involving an epithelial-to-mesenchymal cell transition (EMT), the generation of cancer stem cells (CSCs), invasion of adjacent tissues, and dissemination are fueled by inflammation, which is considered a hallmark of cancer. Chronic inflammation is generated by inflammatory cytokines secreted by the tumor and the tumor-associated cells within the tumor microenvironment. Inflammatory cytokine signaling initiates signaling pathways leading to the activation of master transcription factors (TFs) such as Smads, STAT3, and NF-κB. Moreover, the same inflammatory responses also activate EMT-inducing TF (EMT-TF) families such as Snail, Twist, and Zeb, and epigenetic regulators including DNA and histone modifying enzymes and micoRNAs, through complex interconnected positive and negative feedback loops to regulate EMT and CSC generation. Here, we review the molecular regulatory feedback loops and networks involved in inflammatory cytokine-induced EMT and CSC generation.

## 1. Introduction

The hallmarks of cancer refer to several biological capabilities acquired during the multistep process of tumor initiation and development, which contribute to cancer cell growth, progression, and metastasis. Cancer cell capabilities include sustained chronic proliferative signals through oncogenic mutations and activation of signaling pathways, inactivation of tumor suppressor gene function, resistance to cell death, acquisition of an unlimited growth potential (cell immortality), a sustained supply of nutrients and oxygen by stimulating tumor angiogenesis, and activating local invasion and distant metastasis. The acquisition of these latter capabilities is due to changes in the expression of extracellular matrix (ECM) protein-coding genes, ECM-degrading enzymes, and the activation of an epithelial-to-mesenchymal cell transition (EMT) programme through which transformed epithelial cells acquire mesenchymal cell-like properties enabling them to migrate, invade, and disseminate. Additional cancer cell hallmarks include genome instability, which generates their genetic diversity and the tumor heterogeneity, the acquisition of an inflammatory phenotype, altered cell metabolism, and evasion of immune destruction. In addition to cancer cells, several other cell types are recruited to the developing tumor, creating a certain tumor microenvironment [1].

Cancer onset, development, and progression are controlled by interacting genetic and epigenetic mechanisms, which alter gene expression during carcinogenesis, as determined from the molecular analysis of thousands of human cancers by next generation sequencing (NGS) platforms coupled to chromatin immunoprecipitation techniques (ChIP-sequencing). These studies led to the discovery of inactivating mutations in genes encoding epigenetic regulators and to several alterations in epigenetic mechanisms that can lead to genetic mutations and impaired DNA repair functions [2,3,4].

Epigenetic mechanisms lead to heritable and reversible changes in DNA methylation, histone tail modifications, and noncoding RNAs (ncRNAs) [5]. DNA methylation and histone tail modifications can lead to changes in chromatin structure by altering non-covalent interactions within and between nucleosomes. These modifications are recognized by unique domains of specialized proteins known as chromatin readers, which recruit chromatin remodeling enzymes, which in turn serve as the effectors of chromatin remodeling [2,6,7]. Noncoding RNAs are classified as small ncRNAs and long ncRNAs (lncRNAs), both of which have been implicated in cancer. The small ncRNAs include PIW-interacting RNAs (piRNAs), small nucleolar RNAs (snoRNAs), small interfering RNAs (siRNAs), and microRNAs (miRNAs or miRs), which function in transcriptional and post-transcriptional gene silencing through specific base-pairing with their target mRNAs [8,9]. In contrast, the lncRNAs act as scaffolds or molecular chaperones for chromatin regulators, and their function may be deregulated in cancer [10].

Genetic and epigenetic alterations are required to transform normal cells into malignant cancer cells. Multiple genetic changes in critical genes such as proto-oncogenes and tumor suppressor genes, and disruption of epigenetic regulatory mechanisms alter gene function during the multistep process of carcinogenesis. Cancer cells are characterized by extensive genetic and epigenetic changes compared to their normal counterparts, in a tissue-specific manner. The understanding of interactions between genetic factors and epigenetic regulators is critical as it may influence cancer initiation, development, and progression during the multistep process of carcinogenesis [2,3,4,5,11,12,13,14,15,16]. Inflammatory signaling pathways operate in various cancers, contributing to the induction of EMT, and linking chronic inflammation to tumorigenesis [17,18,19,20,21,22,23,24,25,26,27,28,29,30,31]. Several studies have identified epigenetic regulators impacting on inflammation and EMT in cancer and on the generation of cancer stem cells (CSCs) [16,17,18,19,20,21,22,23,24,25,28,32,33,34,35,36,37,38,39,40], and several molecular circuits have been characterized that regulate these processes [9,37,39,41,42,43,44,45,46,47,48,49,50,51,52,53,54,55,56,57,58]. Importantly, the tumor microenvironment (TME) also influences epigenetic regulation, which contributes to cellular plasticity, the generation of CSCs in tumors, and intra-tumoral heterogeneity [9,16,27,28,29,30,31,33,34,35,36,37,53,54,55,56,57,58,59,60,61,62,63,64,65]. 

The EMT trans-differentiation programme is activated by diverse microenvironmental stimuli including growth factor and cytokine signaling, tumor–stromal cell interactions, and hypoxia. These EMT-inducing signals activate a small set of pleiotropic transcription factors (TFs) that can lead to dynamic and reversible reprogramming of epithelial-to-mesenchymal cell states. However, these oncogenic EMT-inducing transcription factors (EMT-TFs) recruit and interact with epigenetic regulators creating feedback molecular loops that regulate cellular plasticity, and link inflammation with EMT and CSC generation during cancer progression and metastasis [16,22,27,28,29,36,37,41,42,44,45,46,47,48,56,57,65,66,67,68,69,70,71,72,73]. Here, we review how inflammatory cytokines such as TGFβ, TNFα, and interleukins IL-1 and IL-6 regulate signal transducing pathways leading to the activation of master TFs including Smads, NF-κB, and STAT3 and members of the EMT-TF families such as Snail, Twist, and Zeb and epigenetic regulators including DNA and histone modifying enzymes and micoRNAs, through complex interconnected positive and negative feedback loops forming molecular networks, which regulate EMT and CSC generation.

## 2. Regulation of Epithelial-To-Mesenchymal Cell Transition (EMT) in Cancer, and Its Involvement in the Generation of Cancer Stem Cells (CSCs)

While the biological and molecular mechanisms that create primary tumors are relatively well known, the process and mechanisms of metastasis by which cancer cells originating from a malignant primary tumor spread and colonize distant sites, establishing secondary tumors, are less well understood. Metastatic dissemination occurs via a multistep process known as the ‘invasion–metastasis cascade’. This sequence of events is regulated by functions of the cancer cells themselves, the tumor stroma consisting of cancer-associated mesenchymal cells, vascular and immune cells including the tumor-associated microphages (TAMs) and dendritic cells, and its associated TME, which plays an essential role in various stages of carcinogenesis. The ‘invasion–metastasis cascade’ includes the local invasion of primary tumor epithelial cells (carcinoma in situ to invasive carcinoma), entry into the circulation known as intravasation, followed by tumor cell infiltration into distant tissues/organs and invasion in the parenchyma of the infiltrated tissues (extravasation), leading to tissue/organ colonization and formation of metastases [1,22,27,30,31,60,74,75,76,77,78,79,80,81,82]. 

One of the key events in the initiation of tumor cell invasion and metastasis in solid tumors is the activation of the multifaceted EMT programme. EMT is associated with the loss of an epithelial cell phenotype and the acquisition of a mesenchymal-like cell phenotype, which is critical for cancer cell migration, invasion, and metastasis. This phenotypic change is characterized by the functional loss of the epithelial cell marker E-cadherin, which is considered as a hallmark of EMT, and the induction of the expression of mesenchymal cell proteins, including N-cadherin, vimentin, fibronectin, and matrix metalloproteinases (MMPs). Importantly, the EMT is reversible, and tumor cells that display a mesenchymal cell-like phenotype can revert to an epithelial cell phenotype via a process known as mesenchymal-to-epithelial cell transition (MET). A variety of extracellular signals originating from the tumor-associated cells (paracrine signaling) and not from the cancer cells themselves, can contribute to triggering the EMT programme, suggesting that EMT is transient and reversible: In the primary tumor, the cancer cells receive EMT-inducing signals and adopt a mesenchymal cell-like phenotype, but when they disseminate and metastasize at distant sites within the body, they no longer experience the tumor microenvironmental signals responsible for activating the EMT, and they can revert, via MET, to the original epithelial cancer cell phenotype [1,28,29,30,31,60,76,78,79,83,84,85,86,87].

The EMT phenotype is regulated by a small set of pleiotropic oncogenic EMT-TFs and epigenetic regulators [9,36,37,67,68,69,73]. Genetic and epigenetic alterations accumulated within the cancer cell genome during the multistep process of tumorigenesis can create a stable cell-autonomous EMT state [1,36,37,67,68,69,77,78,79]. The best studied example of transcriptional and epigenetic regulation during EMT involves the human *CDH1* (E-cadherin) gene promoter, which possesses several regulatory sequences including three E-boxes that mediate *CDH1* transcriptional repression in mesenchymal cells. Several pleiotropic EMT-TFs have been identified, including the zinc finger TFs of the SNAIL superfamily, such as Snail (SNAI1), Slug (SNAI2), and Smuc (SNAI3); the zinc finger and E-box TFs of the ZEB family, such as Zeb1 (TCF8) and Zeb2 (SIP1); and the bHLH binding proteins E47 and KLF8 (Kruppel-like factor 8), all of which directly repress *CDH1* transcription. The TWIST bHLH TFs (Twist1 and Twist2), the homeobox-binding proteins SIX1 and goosecoid (GSC), the bHLH TFs E2.2, and the forkhead-box protein FOXC2 repress *CDH1* transcription, indirectly [29,36,37,53,54,55,67,68,69]. Moreover, these crucial EMT regulators and *CDH1* transcriptional repressors are direct or indirect (via HIF) downstream NF-κB targets [29,30,88,89,90,91]. 

*CDH1* transcription is also regulated by *CDH1* gene promoter methylation. Tumor cells undergoing transient hypermethylation leading to silencing of *CDH1* transcription are more aggressive, but eventually E-cadherin is re-expressed in metastases due to the demethylation of the *CDH1* gene promoter, highlighting a high degree of epigenetic plasticity in tumor cells [92,93]. While E-cadherin may act pleiotropically to initiate an EMT programme, its down-regulation is not sufficient to elicit a full EMT phenotype [92,93,94], suggesting the operation of additional cooperative mechanisms to silence *CDH1* expression. 

In addition, evidence suggests that EMT is controlled by the interaction between EMT-TFs and epigenetic regulators [3,7,29,37,67,68,69,78,79]. The polycomb group (PcG) proteins form multi-subunit polycomb repressive complexes (PRCs). PRC1 and PRC2 are epigenetic regulators of the expression of *CDH1*. PRC2 contains the histone lysine methyltransferase EZH2 (enhancer of zeste homologue 2), which acts together with the zinc finger protein SUZ12 (suppressor of zeste 12 homologue) to catalyze the trimethylation of K27 on histone H3 (H3K27me3) leading to transcriptional repression. PRC1- and PRC2-mediated silencing of epithelial genes such as *CDH1* transcription involves the initial recruitment of an EMT-TF such as Snail, which binds to the E-box elements of the *CDH1* gene promoter. Snail recruits PRC2 and interacts with EZH2 and SUZ12 to catalyze the addition of a repressive H3K27me3 trimethylation mark that is then recognized by PRC1, leading to silencing of *CDH1* gene transcription [95,96]. 

The regulatory network between EMT-TFs and epigenetic regulators also governs the link between EMT and cancer stem cell (CSC) generation [9,29,36,37,41,42,43,45,46,47,48,53,54,55,57,66,79,96,97,98,99,100,101,102,103]. Genome-wide profiling strategies are used to identify changes in epigenetic modifications during EMT and CSC generation, such as ChIP-seq and ChIP-on-ChIP (ChIP combined with hybridizations on DNA microarray platforms) techniques [104,105,106]. Importantly, because epigenetic alterations such as DNA methylation and histone tail post-translational modifications are reversible, they have become attractive as targets for cancer epigenetic therapy [2,5,107].

Members of the SNAIL superfamily of zinc finger TFs are key inducers of EMT, cell motility, and cell stemness [89,96,101,102,103,108,109,110,111,112]. Snail-mediated recruitment of the histone lysine demethylase LSD1 to target genes can trigger EMT and cancer progression, in conjunction with additional epigenetic modifications. The formation of LSD1–Snail complexes on gene promoters depends on the interaction between the amine oxidase-domain of LSD1 and the SNAG-domain of Snail. LSD1 catalyzes the removal of methyl groups from the H3K4me3 activation mark leading to the loss of transcriptional activation of epithelial genes, including *CDH1* [99,100,113]. LSD1 is highly expressed in several cancer types, displaying mesenchymal gene signatures, and correlates with poor survival [114,115]. Snail-mediated stable silencing also involves the recruitment of the histone methyltransferases G9a (EHMT2) and SUV39H1, which act cooperatively to catalyze the trimethylation of H3K9. The H3K9me3 transcription-repressive mark is required for the recruitment of DNMTs leading to CpG methylation of target gene promoters, stably blocking transcription activity. Snail also interacts with SUV39H1 during TGFβ-induced EMT and mediates silencing of the *CDH1* gene promoter activity by recruiting G9a, SUV39H1, and DNMTs [37,116,117].

Gene expression is also regulated by acetylation and deacetylation of K9 and K14 residues of histone H3, catalyzed by histone acetylases (HATs) and histone deacetylases (HDACs), which can lead to activation or silencing, respectively, of gene transcription. EMT-TFs can repress gene activity through the deacetylation of gene promoters by recruiting HDACs to target gene promoters. During metastasis, EMT-TFs such as Snail recruit and associate with the Mi-2/nucleosome remodeling and deacetylase (NuRD) repressive protein complex containing HDAC1 and HDAC2, which catalyze the removal of acetyl groups from lysine 9 and lysine 14 residues of histone H3 (H3K9/K14), leading to the silencing of the *CDH1* gene promoter [100,109,118]. Twist also associates directly with NuRD, in a different manner than Snail, to silence *CDH1* transcription in mouse and human breast cancer cells [97]. 

Induction of the EMT programme by EMT-TFs–miRNA regulatory circuits leads to the generation of tumor cells with stem cell-like properties that invade adjacent tissues and are resistant to anticancer therapies [1,30,31,38,79,119]. Cancer stem cells (CSCs) displaying a CD44^+^ (high)/CD24^-^(low) cell surface antigen expression profile may act as tumor-initiating cells [120] and contribute to intra-tumoral heterogeneity [29,79]. CSCs migrating away from the primary tumor site enter the circulation to disseminate and localize into distant sites and form metastases. These circulating tumor cells (CTCs) in the blood of patients with advanced primary carcinomas, can extravasate and invade into the parenchyma of distinct tissues/organs. The occurrence of CTCs and disseminated tumor cells (DTCs) correlates with increased tumor aggressiveness and metastatic potential, and decreased time to relapse [29,79].

Several molecular regulatory circuits linking different EMT inducers and epigenetic regulators have been identified. Snail1 can directly repress the transcription of *CDH1* and activate the expression of the ZEB TF genes through different mechanisms [110,121]. Several studies have identified a reciprocal feedback loop between the ZEB family of EMT inducers (Zeb1 and Zeb2) and the miR-200 family members acting as inducers of epithelial differentiation, which mutually control their expression [40,54]. While Zeb1 can induce EMT and a CSC-like phenotype by directly inhibiting the expression of *CDH1* and its own repressor miR-200, miR-200 can promote epithelial cell differentiation by both targeting its own repressor Zeb1 and directly inhibiting the translation of stem cell-associated factors and epigenetic regulators, such as BMI1 [40,54] and SUZ12 [45]. Zeb1, a strong inducer of cancer cell invasion and metastasis in animal models [109], is overexpressed in a large number of human cancer types and is associated with poor prognosis [54,55]. MiR-200 is also overexpressed in certain human cancer types and associated with poor prognosis [55,122]. A possible molecular explanation for these apparently conflicting data is that, although miR-200 down-regulation enhances dissemination, its re-expression may induce MET, which is crucial for metastatic colonization and metastasis [16,55].

While Snail1 is not directly regulated by the ZEB-miR-200 feedback loop, it is part of another reciprocal feedback loop with miR-34. Snail1 can block the transcription of miR-34 family members, whereas miR-34 can suppress the translation of Snail1 [101,102]. However, Snail1 and miR-200c may act antagonistically in EMT induction via a reciprocal regulatory loop [111,123,124]. Thus, the ZEB-miR-200 and the Snail1-miR-34 double negative feedback regulatory loops are linked together via miR-200 to regulate EMT, cell motility, and cell stemness [16,55]. Snail1, a NF-κB target gene [88,108,125], was also shown to suppress p53 by forming a tri-molecular Snail1/HDAC1/p53 complex, which deacetylates activated p53 to promote its proteasomal degradation, leading to an increased expansion of tumor-initiating cells in human breast cancer [103]. Thus, the Snail1-dependent direct repression of both p53 and the p53-miR-34 loop provides another level of control by which canonical NF-κB may suppress p53-induced effects, resulting in EMT, cell motility, cell stemness, and cancer cell metastasis [63,101,102,126,127,128]. 

The members of the miR-200 and miR-34 families are p53-induced miRNAs, which negatively regulate cell plasticity and stemness [53,101,102,111,129], suggesting that p53 controls EMT and cancer cell metastasis through multiple miRNA-dependent processes [36,127,128,129,130]. In tumor cells, p53 is often inactivated by mutations [131], whereas the miR-34a and miR-34b/c genes are silenced by CpG methylation, correlating with metastasis and poor survival [101,127,129,132,133]. As a result, increased Snail1 expression locks cells in a mesenchymal cell-like state and promotes metastasis [63,70,128].

Several different miRNAs are involved in the generation and maintenance of CSCs [134,135], including those regulated by p53 [129] and NF-κB [58,136,137,138]. Clearly, the link between EMT and CSC generation is regulated by the balance of complex interacting TF-miRNA regulatory loops, such as that of the p53-miRNA and the NF-κB–miRNA molecular circuits, which play a crucial role in the control of EMT and cancer cell metastasis [9,39,58,135].

## 3. Inflammatory Molecular Regulatory Circuits Influencing EMT

Inflammation is a physiological mechanism that mediates repair of a damaged tissue, and it is a self-limiting reaction. On the contrary, chronic inflammation has been linked to several human diseases, including cancer [71]. Chronic inflammation induced by an autocrine mechanism or by the TME can promote cancer initiation, development, and progression. It also affects immune surveillance by influencing the crosstalk between tumor infiltrating immune effector cells and tumor cells, through the activation of highly-specific coordinated gene expression programmes, linking immunity to tumor development [17,18,19,20,23,139,140]. Chronic inflammation is now considered a hallmark of cancer [1,20,31].

**Figure 1:**
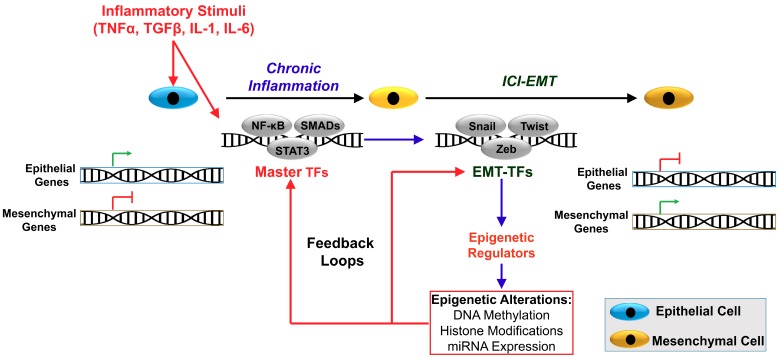
Inflammation is linked to EMT and the generation of cancer stem cells. Inflammatory stimuli initiate signaling transducing pathways leading to the activation of master transcription factors such as Smads, NF-κB, and STAT3. These in turn activate members of oncogenic EMT-TF families such as Snail, Twist, and Zeb, which recruit epigenetic regulators such as DNA and histone tail modifying enzymes, and also affect the expression of noncoding RNAs such as miRNAs. These regulators suppress epithelial cell-specific and activate mesenchymal cell-specific gene expression through interacting positive and negative molecular feedback loops. These inflammatory feedback loops are interconnected generating molecular regulatory circuits and networks to control EMT and CSC generation and maintenance.

Inflammation and EMT are interconnected contributing to cancer progression and metastasis. A number of inflammatory mediators including the cytokines TGFβ, TNFα, IL-1, IL-6, and IL-8, and several chemokines produced by the tumor-associated cells are also EMT-inducers, leading to inflammatory cytokine-induced EMT (ICI-EMT). Hence, the TME modulates EMT [21,22,24,27,38,60,65,71,84,85]. However, ICI-EMT and CSC generation require epigenetic reprogramming involving the creation of molecular regulatory circuits [28,37,41,42,44,46,47,48,56,57,63,66,67,68,69,70,72]. The link between selected inflammatory mediators to EMT and CSC generation, through interacting positive and negative feedback forming molecular regulatory circuits, is discussed below and summarized in Table 1 and Figure 1.

### 3.1. TGFβ

Active TGFβ (TGFβ1, TGFβ2, TGFβ3) dimeric cytokines initiate signaling through a heteromeric receptor complex composed of two type-I (TGFβRI) and two type-II (TGFβRII) transmembrane serine-threonine (Ser/Thr) kinase receptors. In response to TGFβ, the TGFβRII receptor Ser/Thr kinase phosphorylates TGFβRI. TGFβ-induced activation of the TGFβR complex leads to the activation of the receptor-related Smad proteins (R-Smads), Smad2, and Smad3 by direct C-terminal phosphorylation by TGFβRI. The phosphorylated Smad2 and Smad3 form heterotrimers with the common signaling transducer Smad4 and translocate into the nucleus, where they interact and co-operate with transcription factors and epigenetic regulators to activate or repress the transcription of target genes, producing a pleiotropic response [141]. Thus, a functional heterotrimeric complex, Smad2/Smad3/Smad4, controls the transcription of target genes, including those involved in EMT, such as Snail, Slug, and Zeb. In contrast, the inhibitory proteins (I-Smads) Smad6 and Smad7 suppress the activation of the TGFβR-regulated R-Smads in a negative feedback loop [141]. 

TGFβ also induces Snail1 and Snail2 via Smad signaling and IKKα ser/thr kinase, which interacts with Smad3 to control Smad complex formation, leading to *CDH1* silencing in human Panc1 cells [142]. TGFβ-activated Smad2/3 bind to p68 RNA helicase, a component of the miRNA-processing complex Drosha, to facilitate Drosha processing of the primary miR-21 transcript (pri-miR-21) to generate miR-21 in human vascular smooth muscle cells. MiR-21 induces a smooth muscle cell (SMC) contractile phenotype by down-regulating the suppressor PDCD4 (Programmed Cell Death 4), which negatively regulates SMC contractile genes [143].

In addition to the Smad-dependent signaling pathways, TGFβ directly activates several non-Smad signaling pathways including the Ras/Raf/MAPK, the phosphatidylinsitol-3 (PI3) kinase/Akt, NF-κB signaling, and the Rho/Rac1, Cdc42 GTPases, which also play important roles in TGFβ-induced EMT [141,144,145,146].

TGFβ is secreted by the tumor cells and cells of the tumor stroma including CAFs and immune-infiltrating cells. The role of TGFβ during tumorigenesis is complex and paradoxically dual, as it can function as a tumor suppressor in normal and early tumor stages, and as a tumor promoter of cell invasion and metastasis in late-stage cancers, contributing to the malignant progression. This switch in TGFβ function from a tumor suppressor to a tumor promoter is known as the ‘TGFβ paradox’, and it is linked to the initiation of an EMT programme during cancer development and progression [147]. 

TGFβ is a major anti-inflammatory cytokine acting as a tumor suppressor by inhibiting cell proliferation through the increased expression of cyclin kinase inhibitors (CKIs) and inducing apoptosis. TGFβ induces the expression of *CDKN2B*, encoding the CDK4 inhibitor p15^Ink4b^ [148], which is mediated by the binding of a Smad2/3–Smad4–Foxo activator complex to its promoter, leading to dissociation of a Myc–Miz1 repressive complex [149,150,151]. In the absence of TGFβ, a multi-subunit complex consisting of the zinc-finger protein 217 (ZFN217), the co-repressor of RE1-silencing transcription factor complex (CoREST or RCOR1), and the DNA methyltransferase 3A (DNMT3A) binds to the *p15*^Ink4b^ gene promoter, catalyzing the methylation of a CpG island to mediate transcriptional repression [148]. TGFβ stimulation triggers the binding of the Smad2/3–Smad4–Foxo complex to the promoter, which then recruits a DNA excision repair complex composed of the DNA glycosylases, thymine DNA glycosylase (TDG), and methyl-CpG binding domain-4 (MBD4), which remove repressive DNA methylation from the promoter leading to CDNK2B expression. When TGFβ stimulation ceases, the ZFN217-CoREST-DNMT3A prevents Smad2/3–Smad4–Foxo and TDG/MBD4 binding, and returns the promoter to an inactive, methylated, state [148].

TGFβ also acts as a tumor promoter by activating pro-metastatic pathways such as evasion of immune cell function and suppression of inflammation, induction of angiogenesis, and EMT [21,22,141,144,145,147,152,153,154]. The basis for the dual tumor-suppressive and tumor-promoting role of TGFβ is due to several molecular mechanisms. For example, loss of TGFβ-mediated tumor suppression occurs via two distinct mechanisms involving the acquisition of loss-of-function mutations of core TGFβ signaling pathway components such as TGFβRII and Smad4, the epigenetic silencing of TGFβRII, and the decoupling of growth inhibition and apoptosis, and stimulating EMT, cell migration and invasion [15,79,144,147,152,154,155,156,157].

TGFβ-induced EMT is characterized by the reprogramming of specific chromatin domains in the genome. Despite the findings that DNA methylation is unaltered during TGFβ-induced EMT, the epigenetic modifications detected included a global reduction in the heterochromatin repressive mark H3K9me2, an increase in the euchromatin activating mark H3K4me3, and an increase in the transcriptional mark H3K36me3. These epigenetic modifications depended on a functional LSD1, and loss of LSD1 activity has profound effects on EMT-associated cell migration and chemotherapy resistance [158]. 

TGFβ is a strong inducer of the oncogenic EMT-TFs Snail1 and Snail2 (Slug), Zeb, and Twist1 via Smad signaling leading to the suppression of epithelial cell markers and activation of mesenchymal cell-specific gene expression. For example, Snail represses *CDH1* transcription by recruiting epigenetic regulators to *CDH1* gene promoter, as discussed in Section 2 [109,116,117,118,159]. 

LSD1 is an integral protein of the NuRD complex. While the LSD1/NuRD complex transcriptional represses TGFβ signaling, addition of TGFβ induces Snail and LSD1 during EMT of breast cancer cells [114], leading to silencing of E-cadherin expression [99,100], as discussed in Section 2. Interestingly, it was recently shown that nuclear PKCα phosphorylates LSD1, which is required for p65 binding and p65 demethylation, leading to enhanced stability, showing that the PKCα–LSD1–NFκB signaling pathway is crucial for epigenetic control of inflammatory responses and EMT [160]. 

TGFβ induces the expression of JMJD3 (KDM6B), a histone demethylase that activates gene expression by removing histone H3K27me3 repressive marks from chromatin. It was shown that TGFβ-induced JMJD3 expression leads to the expression and activation of Snail1-mediated EMT in normal mouse (NMuMG) and immortalized human (MCF-10A) mammary epithelial cells [161].

TGFβ-induced EMT, cell motility, and cell stemness are associated with reduced cell proliferation of the invading and disseminating tumor epithelial cells, which can be executed by Snail and Zeb TF families by suppressing cell cycle regulators [26,162,163,164,165,166]. The Zeb and the Snail families of EMT inducers are linked in double-negative feedback loops to MET-inducing miR-200 and miR-34 miRNA family members acting as inducers of epithelial cell differentiation, already discussed in Section 2. Thus, TGFβ-induced down-regulation of miR-200 and miR-34 through the Smad-dependent up-regulation of Zeb and Snail, respectively, are crucial to ICI-EMT. TGFβ-induced EMT of mammary epithelia cells involves these linked two double-negative feedback loops: one between the EMT-TF Snail1 and miR-34 and another one between the EMT-TF Zeb1 and miR-200 [50,51]. Thus, these feedback loops drive cell plasticity, enabling switches between the two phenotypic states, EMT and MET [50,51,53,55,101,102,123,124,167]. Importantly, p53 activates the expression of both miR-200 and miR-34 miRNA families, shifting the feedback loops towards a MET programme and proliferation, and from a drug-resistant to a drug-sensitive phenotype [127,128,129,130]. In contrast, NF-κB can suppress p53-mediated effects, resulting in EMT, cell motility, cell stemness, and metastasis [9,58,63,101,102,103].

TGFβ-induced EMT of mammary epithelial cells can result in the up-regulation of SIRT1 deacetylase (NAD-dependent deacetylase sirtuin-1) expression, which catalyzes histone deacetylation and epigenetically silences the *miR-200* gene promoter. SIRT1 and miR-200 are part of a negative feedback loop, as miR-200 targets the 3′ untranslated region of SIRT1 [168].

### 3.2. TNFα

The tumor necrosis factor-alpha (TNFα) is a type-II transmembrane protein with an intracellular amino-terminus, signaling both as a membrane-integrated protein and as a 17 kDa soluble inflammatory cytokine released after proteolytic cleavage. There are two TNF receptors: TNFR1 (or CD120a), which is expressed on most mammalian cells in the body, and TNFR2 (or CD120b), which is primarily expressed on haemopoietic cells. The TNFRs have similar extracellular domains, but TNFR1 is activated by soluble TNFα, and TNFR2 by transmembrane TNFα. TNFRs are also shed and act as soluble TNF-binding proteins, inhibiting TNF activity by competing with the TNF cell surface receptors for free ligand. Transmembrane TNFα functions as both ligand and receptor: soluble TNFRs can bind to the cytokine on the cell surface and generate reverse signaling, leading to immunity and anti-tumor effects. TNFα induces five different types of signals that include caspase-mediated apoptosis pathways, MAPK signaling (ERK, JNK, p38α), and activation of NF-κB signaling [169,170,171].

Activation of NF-κB signaling is initiated by both TNFα receptors, but with a different mechanism. Whereas TNFR2-initiated signaling promotes cell survival, TNFR1-initiated signaling can result in either cell survival or death depending on downstream signaling events and on the cellular context, and these mechanisms and effects are reviewed elsewhere in detail [18,170,171]. The IKK signalsome complex then activates canonical NF-κB signaling, leading to activation and nuclear translocation of p50-p65/RelA heterodimer, influencing the transcription of inflammatory cytokines, anti-apoptotic factors, and cell cycle regulatory genes [172,173,174,175].

TNFα is a major inflammatory cytokine involved in cellular homeostasis, regulating pro-inflammatory responses and various other diverse processes such as cell communication, differentiation, and apoptosis, acting in an autocrine or paracrine manner. A hallmark of TNFα is its ability to promote pro-inflammatory responses, and therefore deregulation of TNFR-mediated signaling is associated with many inflammatory diseases and cancer. TNFα can induce its expression by activating canonical NF-κB. The pro-inflammatory effects of TNFα are mediated by several NF-κB-induced effectors, including the interleukins IL-6, IL-8, and IL-18, chemokines, inducible nitric oxide synthase (iNOS), cyclooxygenase-2 (COX-2), and 5-lipoxygenase (5-LOX), through a regulatory network, which links inflammation to cancer progression and metastasis [169,170,171]. TNFα has both anti-tumor effects mainly by activating apoptotic pathways and pro-tumorigenic effects such as cell survival, angiogenesis, and EMT by activating MAPK and NF-κB signaling pathways leading to inflammatory responses, acting in an autocrine or paracrine manner [169,170,171]. 

TNFα-mediated activation of canonical NF-κB signaling leads to the induction of multiple EMT-TFs, including Twist1 [119,176,177], Snail [89], Snail2 (Slug) [90], and Zeb1/2 [178], and to the repression of E-cadherin expression, and NF-κB-dependent cell migration and invasion in breast cancer cells. NF-κB activation stabilizes Snail1 to promote cell migration and invasion [89]. TNFα also up-regulates TGFβ expression [179] and dramatically accelerates TGFβ-induced EMT [180]. TNFα- or TGFβ-induced EMT in pancreatic cancer cells is dependent on NF-κB signaling [181]. In a three-dimensional (3D) cell culture system in which lung cells were co-stimulated with TNFα and TGFβ, non-small lung cancer (NSCLC) spheroid cultures display elevated expression of EMT-TFs such as Twist1, Snail1, Slug, and Zeb2, in an NF-κB-dependent manner, and are highly invasive and metastatic [182]. TNFα acts synergistically with TGFβ to accelerate the induction EMT in colon carcinoma organoids by a mechanism involving the up-regulation of miR-21, miR-31 [183], and miR-23a in lung epithelial cancer cell lines [184].

Previous studies showed that RelA/p65 can function as a transcriptional repressor via direct recruitment of DNMT1 to chromatin in response to TNFα, leading to methylation of the breast cancer metastasis suppressor 1, *BRMS1*, gene promoter. TNFα-mediated S276 phosphorylation of RelA/p65 by PKA (protein kinase A) or MSK1 (mitogen and stress-activated protein kinase-1) is required for RelA/p65-DNMT1 interactions, chromatin binding of DNMT1, and subsequent *BRMS1* gene promoter methylation, leading to its transcriptional repression in NSCLC cells [185].

TNFα levels inversely correlate with the expression of EZH2 lysine methylase, which controls the epigenetic silencing of specific genes and/or miRNAs by adding an H3K27me3 repressive mark [186], together with SUZ12 recruited by Snail to *CDH1* promoter to transcriptionally silence E-cadherin expression [95,96]. Loss or reduction of EZH2 directly stimulates TRAF2/5 expression to enhance TNFα-induced NF-κB signaling and enhanced inflammatory responses, suggesting that EZH2 acts upstream of NF-κB to antagonize TNFα signaling [187].

MiRNAs are also involved in TNFα-induced EMT. TNFα induces the up-regulation of miR-155 with a dependency on NF-κB activation, which creates a positive feedback loop that in turn enhances NF-κB activity and dictates the intensity of the early stages of inflammatory responses [138,188]. TNFα-promoted EMT requires the induction of miR-105 expression in an NF-κB-dependent manner. It was shown that increased expression of miR-105 in colorectal cancer was associated with an aggressive phenotype [189]. The synergistic action of the positive NF-κB–miR-155 and the negative NF-κB–miR-146a regulatory loops provides an optimal canonical NF-κB activity during inflammation, and eventually leads to the resolution of inflammatory responses [138].

### 3.3. Interleukins

#### 3.3.1. IL-1

IL-1α and IL-1β are structurally similar and have the same functions as they share the common IL-1 type 1 receptor (IL-1R1). *IL-1α* gene expression is induced by Sp1, AP-1, and NF-κB TFs, and IL-1α itself activates canonical NF-κB signaling, creating an autocrine loop. IL-1β expression is induced by AP-1 and NF-κB in immune cells [190,191,192,193]. The cell surface IL-1R1 possesses three immunoglobulin (Ig) domains and one Toll-like/IL-1R (TIR) domain. IL-1 binds to IL-1R1 forming a heterodimer with the co-receptor IL-1R3, which then recruits an adaptor IL-1R-associated kinase (IRAK) and a myeloid differentiation primary response protein 88 (MyD88) via its TIR domain. This leads to the activation of canonical NF-κB signaling pathway and to inflammatory responses in several cell types. Hence, IL-1R1/IL-1R3 heterodimers signal via the MyD88-IRAK-NF-κB pathway [190,191,192,193,194].

IL-1α has a dual function. IL-1α can act as a membrane-bound protein on macrophages by activating the IL-1R1 on adjacent cells through a juxtacrine mechanism. Extracellular IL-1α activates IL-1R1/IL-1R3 and induces a pro-inflammatory response identical to that of IL-1β. Pro-IL-1α precursor with an intact nuclear localization signal (NLS) functions as a transcription factor. During cell apoptosis, pro-IL-1α is tightly bound to chromatin and it is not released. In contrast, in necrotic cells, pro-IL-1α is released and binds to IL-1R1 on adjacent healthy cells to activate an inflammatory response. IL-1α released in this manner is considered an intracellular ‘alarmin’ [190,191,193,195].

IL-1α and IL-1β are amongst the most prominent and potent inflammatory cytokines, together with TGFβ, TNFα, and IL-6, in the TME, and are implicated in cancer-related inflammation. Cancer cells, tumor-infiltrating immune effector cells, and tumor stromal cells express IL-1α, IL-1β, and IL-1R. Both IL-1α and IL-1β are important drivers for the activation of canonical NF-κB-target genes, including inflammatory cytokines and chemokines required for shaping the TME [190,191,192,193]. IL-1α signaling is involved in inflammatory diseases and cancer, driving cancer progression in different ways [195,196]. For example, IL-1R signaling induces the expression of inflammatory mediators, which promote the proliferation and survival of malignant cancer cells [197]. It also induces the up-regulation of stem cell-promoting genes, such as Bmi1 and Nestin, to promote EMT [197,198]. In a chemically-induced skin cancer model, it was shown that IL-1α, acting in an autocrine manner, suppresses the expression of the differentiation markers in mutant Ras-expressing keratinocytes, promoting their neoplastic transformation in a cell-autonomous manner, and the generation of an inflammatory TME via canonical NF-κB-dependent induction of cytokines and chemokines [199]. Similarly, in a pancreatic ductal carcinoma (PDAC) model, a constitutively active *K-Ras* oncogene leads to the activation of canonical NF-κB and inflammatory responses, which further drive NF-κB and STAT3 activation, promoting cell transformation and EMT. In this cancer model, early activation of *K-Ras* through mutation induces IL-1α secretion and p62 feed-forward loops, which further lead to constitutive NF-κB activation, in an autocrine manner, enhancing tumor development, progression, and invasiveness [200,201].

IL-1β also promotes tumor growth and invasiveness [191,192,193]. IL-1β increased tumor progression in mouse tumors, using IL-1β or IL-1R1 gene targeted mice. IL-1β-induced inflammation stimulates angiogenesis by promoting endothelial cell growth, tumor development, cancer cell invasiveness, and lung metastases of melanoma, breast, and prostate human cell lines. IL-1β-induced inflammation also promotes tumor development and invasiveness of 3-methylcholanthrene carcinogen-induced tumors [202,203]. IL-1β has also been shown to induce ICI-EMT, acting alone or in combination with other cytokines including TNFα and TGFβ1 in NSCLC cells, by down-regulating CDH1 expression [204,205]. IL-1β also cooperates with TGFβ3 to promote invasive properties of A549 lung epithelial cancer cells, by inducing several MMPs [206]. Moreover, IL-1β induces ICI-EMT in a 6D breast cancer cell model by activating the IL-1β/IL–1R1/β-catenin pathway and up-regulating Twist1, leading to *ESR1* gene promoter methylation, and to methylation-dependent down-regulation of ERα receptor, linked to tamoxifen resistance [207]. Activation of IL-1β/IL–1RI/β-catenin signaling pathway was shown to activate EMT in a breast cancer cell model through the formation of a TCF/Lef/β-catenin complex leading to the sequential induction of *c-Myc*, *CCDN1*, *Snail1*, *MMP2*, and *BIRC3* (*cIAP2*) expression. This resulted in enhanced cell proliferation, migration, and the acquisition of an aggressive invasive phenotype, including resistance to chemotherapeutic drugs [208,209]. Induction of EMT by IL-1β was also shown to be due to up-regulation of Zeb1 and down-regulation of *CDH1* expression via NF-κB. In addition, it up-regulated the cell stemness factor genes Bmi1 and Nestin and induced CSC self-renewal. These changes lead to increased invasiveness and increased drug resistance [178,210].

IL-1β has also been shown to affect DNA methylation in differentiated colon cancer cells. IL-1β reduced genome methylation of human colon cancer epithelial Caco2 cells and induced CpG demethylation at specific sites in the promoters of the inflammatory cytokine genes *IL-6* and *IL-8*, but not of the *IL10* gene promoter, resulting in their increased expression levels. IL-1β decreases DNMT3b and increases DNMT3a expression, without affecting DNMT1 levels [211].

The Tet methylcytosine dioxygenase 2 (Tet2) is an epigenetic modifier that demethylates deoxycytosine residues in DNA. Tet2-deficient mouse macrophages display increased lipopolysaccharide (LPS)-induced and spontaneous inflammation because Tet2 functions to maintain low levels of IL-1β and IL-6 expression in LPS-treated cells [212]. Tet2 is one of the earliest and most frequently mutated genes causing epigenetic dysregulation in myeloid cancers [213]. IL-1β promotes the recruitment of tumor-promoting myeloid cells in tumors. Activation of the IL-1R–MyD88 pathway leads to the up-regulation of tet2 in tumor-associated macrophages (TAMs) in melanoma mouse models and in patients with melanoma. Tet2 regulates the immunosuppressive programme of TAMs. Genetic inactivation of *tet2* results in macrophage-dependent recruitment of effector T-cells and anti-tumor activity, suggesting that tet2 has a tumor-promoting effect [214]. 

In gastric cancer the survival of tumor cells is increased by IL-1β, induced under conditions of tumor-associated inflammation. IL-1β leads to canonical NF-κB activation, which then targets and up-regulates the tumor-promoting miR-425, which targets the tumor suppressor gene *PTEN* [215]. The expression of the tumor suppressive miR-7 in human gastric cancer inversely correlates with the levels of IL-1β and TNFα, suggesting that miR-7 down-regulation is related to the severity of inflammation and contributes to gastric tumorigenesis [49]. IL-1β was shown to promote the proliferation and migration of NSCLC cells in vitro. IL-1β, acting via the Cox2–HIF1α pathway, suppresses miR-101 expression in lung carcinogenesis. Oncogenic Lin28B was identified as an important effector target of miR-101. Lin28B also suppress the let-7 family of tumor suppressive mRNAs facilitating cellular transformation. Thus, IL-1β up-regulated Lin28B by down-regulating miR-101. IL-1β-mediated repression of miR-101, through a novel regulatory axis involving IL-1β–miR-101–Lin28B, is crucial for inflammation-promoted lung tumorigenesis [216]. IL-1β also leads to the up-regulation of the NF-κB-regulated miR-155 in melanoma cell lines leading to reduced levels of the microphthalmia-associated TF (MITF-M), which drives the expression of many melanocyte-specific differentiation gene products that can be recognized by cytolytic T-cells. Hence, the IL-1β/miR155/MITF-M axis represent a novel mechanism by which melanoma cells escape the immune system in an inflammatory TME [217]. IL-1β/NF-κB signaling stimulates colorectal cancer cell growth via the miR-181a/PTEN axis [218], and it also down-regulates miR-506 expression to promote osteosarcoma cell growth through JAG1 ligand-mediated Notch signaling [219]. Collectively, these studies show how IL-1β regulates miRNA expression in cancer.

#### 3.3.2. IL-6

IL-6 is a multi-functional cytokine produced by several normal and cancer cell types and significantly contributes to inflammatory diseases and cancer [220,221,222,223,224,225].

IL-6 is a pleiotropic cytokine that activates signaling via three different modes. The IL-6 receptor IL-6Rα exists in two forms, as a trans-membrane receptor (mbIL-6Rα) and as a soluble isoform (sIL-6Rα). IL-6 binds to the trans-membrane IL-6Rα (classic signaling) or to sIL-6Rα (trans-signaling) or is presented to neighboring cells via mbIL-6Rα (trans-presentation). Both the low affinity IL-6/mbIL-6Rα or IL-6/sIL-6Rα complexes can recruit the transmembrane-associated signal transducer gp130 (glycoprotein 130) (also known as CD130 and IL-6Rβ) to form a high-affinity IL-6Rα complex that is capable of signal transduction. All three modes of IL-6IL-6Rα/gp130 signaling activate the intracellular JAK-STAT3, JAK-MAPK, and JAK-PI3K signaling pathways. JAK-mediated phosphorylation of STAT3 leads to the formation of STAT3 homodimer complexes, which then translocate into the nucleus, where they influence the expression of IL-6-responsive genes. IL-6 signaling through JAK can also be mediated via Ras/Raf/MAPK, PI3K, or Src/YAP pathways [224,225,226]. One important difference between the IL-6/mbIL-6Rα and IL-6/sIL-6Rα receptor complexes is that the latter can activate autocrine or paracrine IL-6 signaling in cell types expressing gp130 through ‘trans-signaling’, which promotes pro-inflammatory processes. In contrast, the classic IL-6 signaling initiated by the IL-6/mbIL-6Rα/gp130 complex mediates anti-inflammatory effects [221,224,225,227].

IL-6 transcription can be triggered by several inflammatory cytokines including TGFβ, IL-1, and TNFα, and it is activated mainly by NF-κB and STAT3, but also by several other TFs, such as C/EBP, CREB, and AP-1. NF-κB and STAT3 can enhance the expression of IL-6, creating a feed-forward loop implicated in tumor development, progression, and metastasis [222]. Oncogenic signaling pathways such as Ras/Raf/MEK [228,229] or PI3K [230,231] can also enhance the production and secretion of IL-6, which acts in a paracrine manner to promote tumor growth.

IL-6 can either inhibit or promote tumor cell growth in a cell type- and context-dependent manner [232]. Chronic IL-6 signaling plays a crucial role in cancer-associated inflammation in both mouse models and in human disease. IL-6 release is driven by NF-κB, which is aberrantly hyperactivated in many cancers. IL-6 acts both intrinsically on tumor cells and extrinsically on tumor-associated cells within the complex TME to support cancer cell proliferation, survival, angiogenesis, and tumor evasion of immune surveillance. It also regulates cancer–stoma cell interactions and metastatic dissemination and helps to maintain the complex TME [220,221,222,224,233,234,235].

IL-6 signaling can drive cancer progression through the transcriptional activation of target genes involved in cell cycle progression and cell survival [233,234,235,236,237], and via epigenetic mechanisms. IL-6 promotes tumorigenesis by altering DNA methylation in cancer cells, by inducing both the expression of DNMT1 [238] and its nuclear translocation through phosphorylation of its NLS via PI-3K/AKT signaling [239]. IL-6 signaling drives CpG island methylation in promoter regions to inactivate the critical p53 tumor suppressor gene, allowing cancer cells to bypass cell cycle progression checkpoints and also to evade apoptotic signals resulting from DNA damage [239,240,241]. Tp53 represses the expression of IL-6 via direct *IL-6* promoter binding and promoter methylation by inducing DNMT1 [242,243]. TP53 deficiency induces loss of IL-6 promoter methylation, leading to an autocrine IL-6 loop, which induces epigenetic reprogramming to drive cancer cells towards a CSC-like phenotype [238,241,242], via up-regulation of DNMT1 [238,239] by acetylated STAT3 at Lys685 [244]. IL-6 was also found to simultaneously induce CpG promoter methylation of several putative tumor suppressor genes including GATA5, PAX6, and CHFR, inhibiting their expression in oral squamous cell carcinoma cell lines. Simultaneous hypermethylation of multiple tumor suppressor gene promoters by IL-6-mediated signaling leads to epigenetic silencing of tumor suppressor gene expression, suggesting that it may be an important mechanism contributing to chronic inflammation-induced cancer in the oral cavity [245]. Collectively, these studies highlight the requirement for continued IL-6 production in the TME, as chronic IL-6 signaling is required to maintain DNMT1 expression and tumor promoter gene methylation and silencing.

In addition to enhancing cancer cell proliferation and survival, IL-6 induces EMT via JAK-STAT3 or NF-κB pathways, leading to the induction of several EMT-TFs such as Snail, Slug, Twist, and Zeb1, which suppress the expression of *CDH1* via the recruitment of epigenetic regulators. IL-6-induced EMT increases cancer cell migration and invasion through the expression of MMPs in several cancer cell types, [89,112,221,222,246,247,248,249,250,251,252]. Moreover, transcription factors, EMT inducers, and epigenetic regulators, including miRNAs, some of which may be induced during inflammatory responses, operate within molecular interacting feedback loops to affect IL-6-induced cancer cell progression [41,44,47,56,58,63,66,70,90,138,212,215,238,239,240,241,253,254,255,256]. 

One of the most well-defined regulatory circuits linking chronic inflammation and cancer is formed by two distinct but complimentary feedback regulatory loops involving either IL-6, NF-κB, and Lin28, let-7 miRNA or IL-6, NF-κB, STAT3, miR-21, miR-181b-1, and the tumor suppressor genes *PTEN* and *CYLD* [41,44]. Transient induction of the *src* oncogene in immortalized human mammary epithelial cells, MCF-10A, triggers an inflammatory response, through the secretion of IL-6, which is mediated by canonical NF-κB activation. NF-κB, in turn, up-regulates the expression of Lin28 RNA binding protein, which binds to the tumor suppressor let-7 pre-miRNA, leading to the inhibition of the expression of mature let-7 miRNA [257]. Since let-7 normally targets IL-6, its down-regulation leads to IL-6 overproduction. IL-6 signaling activates STAT3 and also NF-κB, maintaining the cycle of events and establishing a feedback loop, which drives and maintains human breast cells in a transformed state. Hence, an inflammatory signal from non-transformed, spontaneous, immortalized cells to cancer cells initiates a positive feedback loop involving IL-6/NF-κB/Lin28/let-7 miRNA and STAT3 activation [41,42].

NF-κB and STAT3 are the main transcription factors that modulate and are also regulated by inflammatory responses and can influence each other through multiple feedback mechanisms that involve miRNAs [9,56,58,70,253,256]. A complimentary feedback loop is formed with the aid of STAT3, which is also activated by IL-6, which maintains NF-κB in an active state. STAT3 induces miR-21 and miR-181b-1 expression, which target the tumor suppressor genes *PTEN* and *CYLD,* respectively, leading to canonical NF-κB activation [44]. Collectively, IL-6, through the synergistic action of NF-κB and STAT3 TFs, together with the functions of miR-21, miR-181b-1, and let-7, establishes a feedback mechanism to sustain inflammatory signals to directly link chronic inflammation and cancer. In addition to NF-κB, STAT3 can also be further up-regulated by this feedback loop, since STAT3-mediated induction of miR-181a/b can also lead to the activation of IL-6/STAT3 signaling [44]. Other studies revealed the existence of a negative feedback loop between STAT3 and NF-κB through miR-146b. In this case, STAT3 induces miR-146b, which targets NF-κB, reducing IL-6 secretion, and leading to the resolution of inflammation. Reduced IL-6 production is the final step of a negative feedback loop, since IL-6 induces STAT3 activation, contributing to chronic inflammation [138,256]. Collectively, these studies have established the critical interplay between NF-κB and STAT3 in the regulation of inflammation-induced cancer. Moreover, these molecular regulatory circuits have shown that mammary tumor cells are able to survive through autocrine IL-6 production, and independently of the paracrine release of IL-6 by the tumor stromal cells. 

IL-6-mediated inflammation is also linked to Tet2, which catalyzes the conversion of 5-methylcytocine to 5-hydroxymethylcytosine (5hmC) and actively demethylates DNA. It was shown that IκBζ, an IL-6-specific TF and a member of the IκB family of NF-κB inhibitors, mediates specific targeting of Tet2 to the *Il6* promoter, which then recruits HDAC2 to repress *Il6* transcription via histone deacetylation. Loss of Tet2 results in the up-regulation of several inflammatory cytokines, including IL-6, at late phase in response to LPS [212]. Tet2 is a direct target of the let-7adf miRNAs, which are physiological inducers of IL-6 production [258]. 

Another regulatory loop that connects inflammation with the various stages of liver carcinogenesis is the one involving hepatocyte nuclear factor 4α (HNF4α), IL-6, and miRNAs. HNF4α is a TF that takes part in two distinct feedback loops that occur in normal and cancer cells, respectively, consisting of IL-6R; the miRNAs miR-24, miR-124, and miR-629; and STAT3. In normal cells, HNF4α up-regulates miR-124, which targets the IL-6 receptor, leading to suppression of inflammation-induced IL6-STAT3 signaling. In cancer cells, inhibition of HNF4α leads to an inflammatory-response-mediated regulatory feedback loop initiating and maintaining hepatocellular carcinogenesis via STAT3-mediated induction of miR-24 and miR-629 expression. In this mechanism, the first step involves activation of IL-6/STAT3 signaling in the absence of HNF4α-miR-124, and the second step involves IL-6/STAT3 mediated induction of miR-24 and miR-629 expression. Both miR-24 and miR-629 target HNF4α, further sustaining the activity of inflammatory signaling, possibly contributing to ICI-EMT and hepatocellular carcinogenesis. The main characteristic of the HNF4α feedback loop in liver cancer is that overexpression of a positive factor such as miR-24 and miR-629 or inhibition of a negative factor such as HNF4α and miR-124 transforms immortalized hepatocytes, suggesting that this feedback circuit can be influenced at any step [47].

In relation to colon cancer, it was shown that IL-6/STAT3-driven EMT in a colorectal cancer model requires miR-34a suppression. Treatment of human colorectal cancer (CRC) cells with IL-6 leads to the activation of STAT3, which directly represses the expression of the tumor suppressive p53-regulated miR-34a gene via a conserved STAT3-binding site in its first intron. The IL-6R mediating the IL-6-dependent activation of STAT3 was identified as a conserved, direct target of miR-34a [57,63,70,128]. Importantly, miR-34a suppresses EMT by directly targeting Snail1, which in turn directly suppresses *CDH1* transcription [101,102]. Repression of miR-34a was required for IL-6-induced EMT, invasion, and metastasis. The resulting IL-6R/STAT3/miR-34a feedback loop operates in primary colorectal (CRC) tumors and it is also present in CRC, breast, and prostate cancer cell lines and is associated with a mesenchymal cell-like phenotype. Moreover, colitis-associated intestinal tumors of miR-34a-deficient mice progress to invasive colon carcinomas and display elevated levels of phosphorylated STAT3, IL-6R, and Snail expression. The IL-6/STAT3/miR-34a feedback loop operating in primary CRC tumors is also associated with lung metastases in patients. p53 activation, often through gain of function mutations in CRC cells interferes with IL-6-induced EMT, cell migration, and invasion via miR-34a-dependent down-regulation of IL-6R expression. Collectively, these studies showed that p53-dependent expression of miR-34a suppresses tumor progression by inhibiting the IL-6R/STAT3/miR-34a feedback loop [57,63,70,128]. This EMT-inducing feedback loop may act antagonistically with the Zeb-miR200 feedback loop, which promotes MET, thereby creating a double negative molecular circuit, which are linked together via miR-200 to regulate EMT, cell motility, and stemness [9,16,20,55,70,101,102,124].

Nanostring analysis identified nine miRNAs in human lung cancer cell-derived exosomes with the oncogenic miRNAs miR-21, miR-27b, and miR-29a being expressed at significantly higher levels [254]. MiR-21 and miR-29a can reach and bind as ligands to members of the TLR receptor family, in both murine (TLR7) and human (TLR8) in immune effector cells leading to TLR-mediated canonical NFκB activation and secretion of the pro-inflammatory cytokines TNFα and IL-6, which then can stimulate tumor growth and metastasis [254]. Thus, miRNAs in lung cancer-derived exosomes may act in a paracrine manner to activate TLRs in the surrounding macrophages to secrete inflammatory cytokines, influence the TME, and promote tumor growth, invasion, and metastasis [254,259]. 

### 3.4. Converging Roles of Inflammatory Cytokines during ICI-EMT

Inflammation is linked to cancer development and progression involving EMT, cell invasion, and metastasis. Inflammation-induced EMT is regulated by various mechanisms involving the initiation of cytokine-induced signal transducing pathways leading to the activation of master transcription factors, EMT-TFs, and epigenetic regulators generating positive and negative molecular feedback loops (Figure 1). For example, TGFβ activates Smad2/3–Smad4 transcription factors, MAPK, and NF-κB signaling; TNFα and IL-1 operate mainly via activation of the canonical NF-κB signaling pathway; and IL-6 activates STAT3 and NF-κB signaling. Inflammatory cytokine-driven EMT mediated by Smad, STAT3, and NF-κB TFs leads to the activation of oncogenic EMT-TFs, which recruit epigenetic regulators in the *CDH1* gene promoter resulting in the silencing of E-cadherin expression. The inflammatory mediators also influence the expression of several miRNAs, which positively or negatively regulate inflammatory cytokine signaling through interacting feedback loops forming molecular regulatory networks [9,16,41,44,47,48,55,58,63,70,87,101,102,128] (Table 1 and Figure 1).

## 4. Epigenetic Regulation of CSC Generation: The Role of Inflammation and the Tumor Microenvironment

Inflammatory cytokine-induced EMT also leads to the generation of CSCs [38,86,87]. The EMT programme induces CSCs at the invasive front of tumors and initiates the dissociation of cancer cells and CSCs from primary carcinomas, which subsequently migrate and disseminate to distant sites in the body. These CSCs are characterized by the expression of a specific cell surface marker profile, such as CD44^high^CD24^low^ in breast CSCs or CD133^+^ marking CSCs in various tumor types [1,29,78,79,83,87,260]. EMT and CSC generation is controlled by molecular regulatory feedback loops between EMT-TFs and epigenetic regulators [9,28,29,36,37,39,40,41,42,43,44,45,46,47,48,50,51,52,53,54,55,56,57,63,66,70,79,87,96,97,98,99,100,101,102,128].

TGFβ signaling via Smad2/3 induces EMT, which leads to CSC generation [156]. One mechanism involves the TGFβ-induced expression of Snail, which recruits DNMTs leading to CpG methylation and the histone lysine methyltransferases G9a (EHMT2) and SUV39H1 to *CDH1* gene promoter, which cooperatively catalyze the trimethylation of H3K9 leading to the loss of E-cadherin expression. This leads to increased cell migration and invasion in vitro and lung metastasis in mice [116,117]. Another mechanism involves arginine methylation of Smad7, an inhibitor of Smad3, by the arginine methyltransferase 1 (PRMT1), promoting TGFβ-induced EMT and CSC generation [261]. TGFβ also up-regulates miR-181a, which targets TIMP3 to promote breast cancer cell metastasis [262] and hepatocarcinogenesis [263].

The microRNA-520/373 family functions as a tumor suppressor in ER-negative breast cancer by targeting NF-κB p65/RelA and also TGFβ signaling by directly suppressing TGFBR2 and several Smad-dependent metastasis-promoting genes [264].

TGFβ-induced EMT in mammary epithelial cells leads to the induction of the JMJD3 (KDM6B), which is recruited to the *Snail* gene promoter to catalyze the removal of a repressive H3K27me3 mark, thereby inducing Snail expression, which in turn activates mesenchymal cell-specific genes. JMJD3 expression was found to be significantly increased in invasive breast carcinoma compared to normal breast tissues [161].

The most important mechanism of TGFβ/Smad-induced EMT leading to CSC generation involves the two double-negative feedback loops, Zeb/miR-200 and Snail-miR-34, which regulate both EMT and CSC generation, but also MET [50,51,53,55,101,102,123,124,128,167]. Studies also showed that TGFβ-driven EMT of mammary epithelial cells and CSC generation is controlled by an Akt/miR-200/E-cadherin axis [43].

IL-6 stimulates the growth of CSCs. IL-6 treated primary human mammospheres and human breast cancer cells grown in suspension (three-dimensions) produces multicellular spheroid structures containing stem/progenitor cells dependent on Notch-3 signaling [265]. Moreover, IL-6 triggers an autocrine/paracrine Notch-3/JAG-1 loop to further enhance self-renewal of stem/progenitor cells in the mammary gland [265]. CSCs recovered in small numbers from several primary tumors or xenografts including breast, liver, and lung secrete IL-6, which acts in an autocrine manner leading to their enhanced cell proliferation and survival [66,72,266,267]. Moreover, cancer-associated fibroblasts isolated from breast cancer metastatic sites enhance cancer cell growth and invasiveness in an IL-6-dependent manner [268]. It was shown that the activation of an IL-6/STAT3/PTEN/NF-κB inflammatory feedback loop mediates trastuzumab resistance in HER^+^ breast cancer cells by expanding the CSC population. The IL-6/JAK1/STAT3 signaling pathway is also important and sufficient in the conversion of non-CSCs into CSCs [66,269] through regulation of Oct4 expression in human breast cancer lines [270]. IL-6 also seems to enhance the ability of breast cancer cells (tumor self-seeding) to survive, seed, and colonize distant sites, thereby contributing to tumor progression and metastasis [271]. Knockdown of the tumor suppressors *p53* and *PTEN* collaborated in the secretion of IL-6 and activation IL-6/STAT3/NF-κB signaling in immortalized human mammary epithelial MCF-10A cells, leading to the induction of EMT and metastatic CSCs [272].

IL-6-mediated induction of cancer cell stemness is also controlled by epigenetic processes [41,42,44,45,46,63]. It was shown that cancer cells expressing mutant p53 secrete more IL-6 that their wild-type (wt) counterparts because wt-p53 inhibits the binding of NF-κB, C/EBP, and CREB TFs to the *IL-6* gene promoter [242]. Subsequent studies showed that p53 recruits DNMT1 methylase to target promoters [243], and that p53 deficiency induces the loss of IL-6 promoter methylation, initiating an autocrine IL-6 loop [241]. IL-6 signaling up-regulates DNMT1 and maintains p53 gene promoter methylation [238,239,273]. It also leads to STAT3 activation and acetylation at Lys685 in tumor cells, which is crucial for methylation of tumor-suppressor gene promoters, resulting in their inactivation [244]. Hence, the generation of this autocrine IL-6 loop induces an epigenetic reprogramming that drives cancer cells towards a stem cell-like phenotype [238,241].

IL-6 also promotes cell stemness and aggressiveness of glioblastoma tumors by suppressing miR-142-3p expression. IL-6 promotes hypermethylation of a Sp1-binding site in the *miR142-3p* gene promoter, preventing binding of Sp1 and inhibiting miR-142-3p expression. However, miR-142-3p also suppresses IL-6 expression by targeting the IL6 3′-UTR, thereby forming an IL-6/miR-142-3p feedback loop. MiR-142-3p inhibited tumor growth in in vivo xenografts in mice. In addition, glioblastoma patients displaying *miR-142-3p* gene promoter methylation show poor survival [274]. IL-6/STAT3 directly induces the expression of the p53-regulated tumor suppressive miR-146a. This then inhibits NF-κB-dependent production of IL-6, STAT3 activation, and IL-6/STAT3-driven cell migration and invasion of breast cancer cells, thereby establishing an IL-6/STAT3/NF-κB/miR-146a negative feedback loop. While expression of miR-146a is increased in normal mammary epithelial cells, it is decreased in primary breast cancers, relieving miR-146-induced inhibition of STAT3 expression, which could exert its oncogenic functions in breast cancer cells [256].

Previous studies showed that *src* oncogene-induced transformation of spontaneously immortalized MCF-10A breast epithelial cells and activation of the NF-κB/IL6-STAT3/lin28/let-7 feedback loop was required for EMT and the enrichment of the tumor-initiating cells, or the CSC population with self-renewal capacity. The inflammatory loop was more active in this population compared to cells without self-renewing capacity [41,42]. During this process of CSC generation, interactions between miR-200b and Suz12, a subunit of PRC2, are required for CSC generation. MiR-200b directly targets Suz12, and loss of miR-200 during CSC generation increases Suz12 expression and binding, trimethylation of H3K27, and PRC2-mediated repression of *CDH1* gene transcription. Loss of miR-200 results in increased Zeb1 and Zeb2 levels, leading to suppression of *CDH1* expression and EMT induction [177]. This stable epigenetic silencing of *CDH1* expression was critical for maintaining a CSC state [45,46]. Activation of the src oncoprotein in MCF-10 cells also identified additional miRNAs, including miR-145 and other members of the miR-200 family, such as miR-200a and miR-200c, that are repressed in CSCs [45]. Importantly, IL-6/STAT3-mediated inhibition of miR-200c during transformation and EMT of breast [275] and lung [276] epithelial cancer cells has also been demonstrated by others.

## 5. Concluding Remarks

Inflammatory cytokines such as TGFβ, TNFα, and interleukins including IL-1 and IL-6 initiate signal transducing pathways leading to the activation of master transcription factors such as Smads, NF-κB, and STAT3. These then activate downstream oncogenic EMT-TFs such as SNAIL, TWIST, and ZEB family members, which in turn recruit epigenetic regulators such as DNA and histone tail modifying enzymes, which also affect the expression of noncoding RNAs such as miRNAs. MiRNAs influence the activity of both master TFs and EMT-TFs, shaping cytokine signaling through several positive and negative feedback loops. These inflammatory feedback loops are interconnected, generating molecular regulatory circuits and networks to control EMT and CSC generation and maintenance. As these molecular regulatory circuits are generated by the activation of inflammatory cytokine signaling, it would be challenging to identify specific protein kinases regulating the activity of TFs and the expression of miRNAs involved in novel molecular feedback loops.

## Figures and Tables

**Table 1:** Overview of major epigenetic alterations induced by cytokines and associated with EMT and CSCs.

Cytokine	Master TFs	Epigenetic Mechanism	Effect
**TGFβ**	Smads	Induction of Snail, Slug, and Zeb TFs up-regulates mesenchymal genes through reprogramming of specific chromatin domains (reduction of histone methylation by LSD1 and KDM6B/JMJD3 demethylases)	EMT and cancer progression
**TGFβ**	Smads	Snail- and Zeb-dependent down-regulation of miR-34 and miR-200 (double negative feedback loops) SIRT1-dependent miR-200 down-regulation by histone deacetylation (negative feedback loop)	Induction of EMT and inhibition of MET
**TGFβ**	Various TFs	Several Smad-independent alterations, through MAPKs, PI-3K, and ΝF-κB pathways	EMT and cancer progression
**TGFβ**	Smad2/3–Smad4–Foxo	Reduced proliferation through induction of CDKN2B/p15^ink4b^, CDK4 inhibitor (removal of DNA methylation on CDKN2B promoter)	Tumor suppression
**TNFα**	NF-κB	Induction of ΝF-κB-regulated proteins (IL-6, IL-8, IL-18, iNOS, COX-2, 5-LOX, etc.)	Inflammation-dependent cancer development
**TNFα**	NF-κB	Induction of Twist1, Snail, Snail2, and Zebs leading to E-cadherin down-regulation and mesenchymal genes induction	EMT, migration and invasion
**TNFα**	NF-κB	DNMT1-dependent down-regulation of metastasis suppressor BRSM1	Invasion and metastasis
**TGFβ + TNFα**	Smads + NF-κB	Induction of EMT TFs leading to epigenetic alterations that induce mesenchymal genes and several oncomiRs (miR-21, miR-31, miR-23a, etc.)	EMT and cancer progression
**IL-1**	NF-κB	Expression of several inflammatory mediators (including IL-1, leading to an autocrine loop)	Inflammation-dependent cancer development
**IL-1α**	NF-κB + STAT3	Induction of stem-cell associated genes bmi-1 and Nestin	EMT and CSC development
**IL-1β**	β-catenin	Expression of c-MYC, CCDN1, Snail1 and MMP2, and BIRC3. Twist1-dependent methylation of ESR1 gene promoter	EMT and cancer progression Resistance to chemotherapy
**IL-1β**	NF-κB	Induction of miR-181 or down-regulation of miR-506	Cancer progression
**IL-1β + TNFα + TGFβ**	Several	Induction of inflammatory genes and several MMPs	EMT, migration and invasion
**IL-6**	STAT3	Induction of cell cycle regulators (E2Fs, JunB, c-Fos, etc.) and metabolic regulators (mTORC1)	Cancer progression
**IL-6**	-	DNMT1 dependent DNA methylation of tumor suppressor gene promoters (CHFR, GATA5, and PAX6), including TP53 (negative feedback loop)	Cancer progression and CSC formation
**IL-6**	ΝF-κB + STAT3	Induction of EMT-TFs Snail, Slug, Twist, and Zeb (down-regulation of E-cadherin and up-regulation of mesenchymal genes and MMPs)	EMT, migration and invasion
**IL-6**	ΝF-κB + STAT3	Feedback loops involving IL-6, ΝF-κB, Lin28, and let-7 miRNA or IL-6, ΝF-κB, STAT3, miR-21 and miR-181b-1, and PTEN and CYLD	Cancer progression
